# Fibroadenoma of the breast in a South African population -a pilot study of the diagnostic accuracy of fine needle aspirate cytology and breast ultrasonography

**DOI:** 10.4314/ahs.v18i2.11

**Published:** 2018-06

**Authors:** Sumana Pillay, Shalen Cheddie, Yoshan Moodley

**Affiliations:** 1 University of KwaZulu-Natal College of Health Sciences, Department of Surgery; 2 Madadeni Hospital; 3 University of Kwazulu-Natal, Discipline of Anaesthetics and Critical Care

**Keywords:** Fibroadenoma, diagnosis, breast ultrasonography, fine needle aspirate cytology

## Abstract

**Background:**

The triple assessment of clinical breast exam (CBE), fine needle aspirate cytology (FNAC) and breast ultrasonography (US) is used in many settings for the diagnosis of fibroadenoma (FA). The diagnostic accuracy of FNAC and US for FA in South African (SA) women with palpable breast masses (PBM) is unknown.

**Objective:**

To report the diagnostic accuracy of FNAC/US for FA in SA women with PBM.

**Methods:**

We conducted a retrospective pilot diagnostic study of 91 women who presented with PBM to a SA regional academic hospital. Data for CBE, US, unguided FNAC, and open biopsies was collected from study participant medical records and analyzed using diagnostic accuracy tables.

**Results:**

A total of 57/91 (62.6%) study participants had uninterpretable FNAC results. No study participants had uninterpretable US results. The overall diagnostic accuracy of FNAC for FA was 36.3% (95% Confidence Interval - CI: 27.1–46.5%). The overall diagnostic accuracy of US for FA was 83.5% (95% CI: 74.6–89.8%).

**Conclusion:**

The yield of interpretable test results for FNAC was poor in our study. The diagnostic accuracy of US for FA appears to be superior to that of FNAC. Omission of FNAC from the triple assessment in our setting should be considered.

## Introduction

Fibroadenoma (FA) is the most common palpable benign breast lesion in African women^1^–^8^. The triple assessment is used to investigate for FA and consists of clinical breast examination (CBE), breast ultrasonography (US), and fine needle aspirate cytology (FNAC)^9^,^10^. This three pronged approach of investigation results in a diagnostic accuracy approaching 100%^10^. However, discordant or uninterpretable results in one of the three investigations would require that a core needle biopsy (CNB) and subsequent histological analysis be performed^10^. The FNAC test may yield uninterpretable results for 22–28% of specimens tested^10^. This in turn might result in unwarranted CNB, with additional economic consequences for healthcare systems and psychological consequences for afflicted patients^11^,^12^. Assessing the accuracy of FNAC and US for the diagnosis of FA in a South African (SA) setting would have important future implications related to health care expenditure and resource utilization for benign breast disease in SA, and how FA is managed in SA women. The objective of this pilot study was to provide a report of the diagnostic accuracy of FNAC and US for the detection of FA in SA women.

## Methods

### Study design, setting, and study population

This was a retrospective pilot diagnostic accuracy study conducted at the King Edward VIII Hospital (KEH) in Durban, SA between 01 January 2012 and 31 December 2014. The King Edward VIII hospital is a regional academic hospital which provides health care services to the population residing in parts of the eThekwini municipality. The study population consisted of a consecutive/convenience sample of 91 female participants with palpable benign breast masses (PBM) who presented to the surgical out-patient clinic at KEH during the aforementioned study period (ie. we operated on every consecutive patient who attended surgical out-patient clinic with a PBM). All participants had a “triple assessment” performed. As per conventional practice for the triple assessment, the results of FNAC and US (as well as CBE) were taken together into consideration. Image-guided CNB is the international standard for obtaining histological samples for the diagnosis of breast disease. However, all patients in our study population opted to have the masses excised through an open surgical procedure due to cosmetic reasons or fears of malignancy. Furthermore, the limited availability of radiological staff and equipment during the study period rendered image-guided CNB unfeasible in our specific setting. Therefore, open biopsy and subsequent histological analysis (OBHA) was used as the confirmatory diagnostic method in our study.

### Data collection

Data related to patient age, CBE, US, FNAC, and OBHA were collected for each of the participants included in this study using a formal data collection instrument. With regard to CBE, the number, size, and location of the PBM was recorded for each participant. Data for US and FNAC were broadly classified according to the reporting categories used by the UK National Health Service Breast Screening Programme (UK-NHSBSP)^13^. The US and FNAC procedures were performed via an unguided approach by trainee radiologists/physicians, under the supervision of consultant radiologists/physicians. While the image guided approach is the recommended approach for FNAC in most settings, the availability of resources (functioning US machines and radiologists to assist with these procedures) at our surgical out-patient department was limited during the study period. Therefore, FNAC in our setting had to be performed through the unguided approach. Conventional ultrasonographic and cytologic criteria were used to diagnose suspected FA. The open biopsy procedures were performed by trainee surgeons under the supervision of consultant surgeons. Tissue specimens from open biopsies were sent to specialist pathology laboratories for analysis. Conventional histological criteria were used to confirm FA. Results from the histological analysis were summarized into three categories: FA, other benign lesion, or malignancy. In our resource-limited setting it took up to a month after CBE to have a US performed, with FNAC performed up to a week after US. The FNAC specimens took up to three weeks to be analyzed and reported. The time period between receiving a FNAC result and performing an open biopsy was four to six weeks. Histological findings from the open biopsy were made available one month following the procedure. Clinical information and results from the open biopsy were made available to attending physicians. Clinical information and findings of the US/FNAC were not available to the pathologists performing the histological assessment. Data were transferred from the data collection instrument to a Microsoft Excel® spreadsheet for subsequent statistical analysis.

### Sample size and statistical analysis

The study population consisted of 91 female participants. This sample size was deemed sufficient for this pilot diagnostic accuracy study as it exceeds the minimum sample size recommended for pilot studies in medical settings^14^. There were no participants in the study population with missing test results/reports. We first conducted a descriptive analysis of age, CBE, US, FNAC, and OBHA results for the study population. Results for this descriptive analysis are presented as medians (with interquartile range) or frequencies and percentages. The initial descriptive statistical analysis was performed in Microsoft Excel® using standard formulas/approaches in the software program. Measures of diagnostic accuracy were computed using the conventional 2x2 table approach where there were no uninterpretable diagnostic test results^15^. Participants with uninterpretable diagnostic tests results were not excluded from the data analysis. Rather, the method recommended by Simel and colleagues^16^ often described as the “3x2 table” approach, was used to compute measures of diagnostic accuracy where there were uninterpretable diagnostic test results. Sensitivity, specificity, overall test yield, likelihood ratios, and diagnostic accuracy in the format recommended by Simel and colleagues^16^, with 95% confidence intervals (95% CI). The overall reporting of our study is in accordance with the 2015 STARD guideline^17^.

### Study ethical approval

The study was given full ethical approval by the Biomedical Research and Ethics Committee of The University of KwaZulu-Natal (Protocol: BE053/13).

## Results

### Description of the study population

The median age of the study population was 21.0 (18.0–26.0) years old. Approximately 60% (n=55/91) of study participants had PBM measuring ≥3cm in size. The majority of study participants presented with a single PBM (n=84/91, 92.3%). Just over half of study participants had the PBM located on the right breast (n=48/91, 52.7%). When stratified according to the reporting categories used by the UK-NHSBSP, 90 study participants (98.9%) had findings of benign/probable benign lesions on US and one participant had findings on US suspicious of malignancy (1.1%). No OBHA tests were missing and no OBHA results were inconclusive. The main reason for why masses were biopsied or removed in our young study population was that these masses were found to be increasing in size, or were already of considerable size (≥3cm in diameter) and had a negative impact on quality of life in afflicted patients. All histological specimens (n=91/91, 100.0%) were diagnosed as benign breast disease, with 84.6% (n=77/91) of study participants being diagnosed with FA on histology. When FNAC results were stratified according the UK-NHSBSP reporting categories, 62.6% (n=57/91) of participant specimens were classified as inadequate, and the remaining 37.4% (n=34/91) of FNAC specimens were classified as benign/probably benign lesions.

### Description of FNAC results

**Figure 1:**
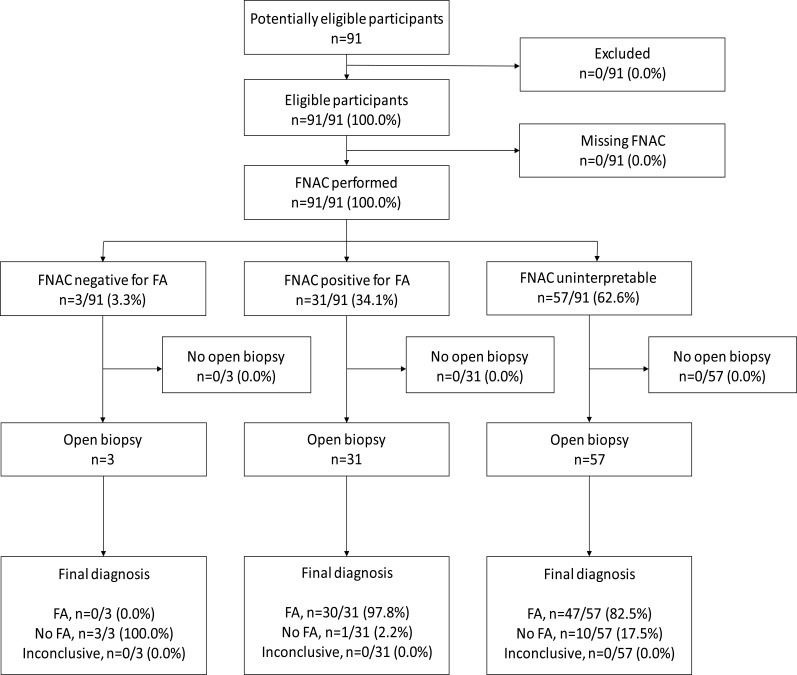
Study participant flow diagram — FNAC for the diagnosis of FA

The study participant flow for the FNAC test is shown in Figure 1. All study participants had a FNAC test performed. An interpretable FNAC result was obtained for 37.4% of study participants. Approximately 3.3% (n=3/91) of study participants tested negative for FA on FNAC, and 31.4% (n=31/91) tested positive for FA on FNAC. The remaining 62.6% (n=57/91) of study participants had uninterpretable FNAC test results. All three study participants who tested negative for FA on FNAC did not have FA on OBHA. Of the study participants who tested positive for FA on FNAC (n=31), 97.8% (n=30/31) had FA confirmed on OBHA, with the remaining participant (2.2%) diagnosed with a cyst. Of the participants with uninterpretable FNAC test results (n=57), 82.5% (n=47/57) were diagnosed with FA on OBHA, and the remaining 17.5% (n=10/57) were diagnosed with cystic lesions on OBHA.

### Description of US results

**Figure 2:**
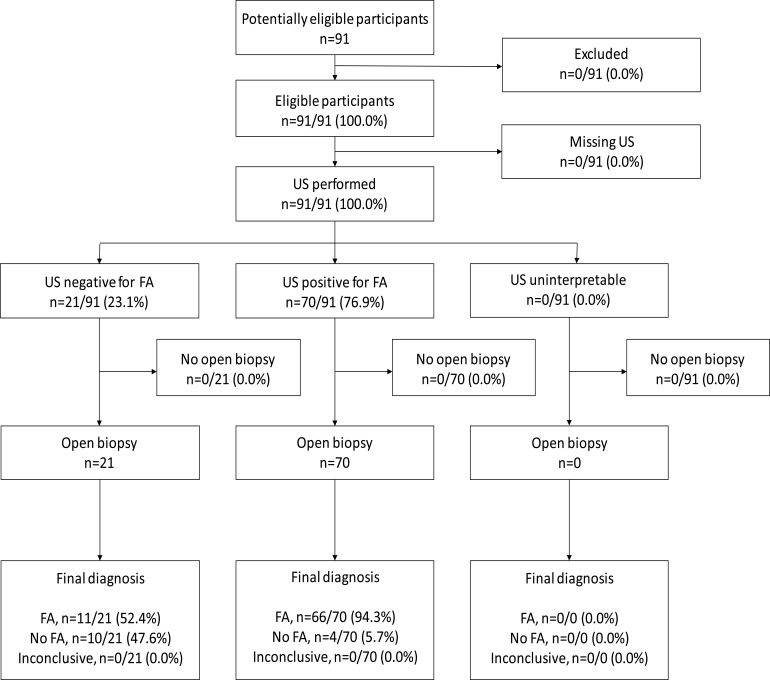
Study participant flow diagram — US for the diagnosis of FA

The study participant flow for the US test is shown in Figure 2. As with FNAC, all 91 study participants had the US test performed. All study participants had interpretable US results. Approximately 23.1% (n=21/91) of study participants tested negative for FA on US, while the remaining 76.9% (n=70/91) of study participants tested positive for FA on US. All study participants with US tests had OBHA performed with interpretable results. Almost half of the study participants who tested negative for FA on US (47.6%, n=10/21) did not have FA on OBHA. Of the study participants who tested positive for FA on US (n=70), 94.3% (n=66/70) had FA confirmed on OBHA, with the remaining four participants (5.7%) diagnosed with a cystic lesion on OBHA.

### Cross-tabulation of test results and measures of diagnostic accuracy

**Figure 3:**
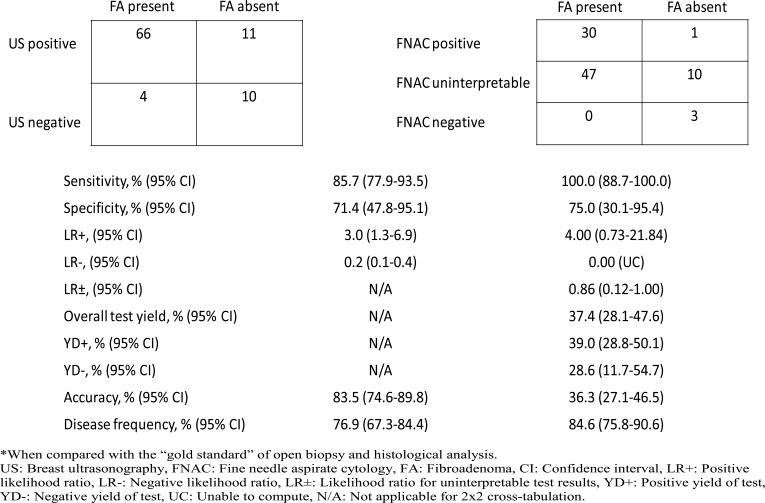
Cross-tabulation of US and FNAC test results by histology findings and estimates of diagnostic accuracy*

Cross-tabulation of test results, as well as measures of diagnostic accuracy are shown in Figure 3. A large proportion of the study population had uninterpretable FNAC test results. Therefore, the cross-tabulation and associated measures of diagnostic accuracy for the FNAC test results are presented as a 3×2 diagnostic accuracy table. The sensitivity and specificity of FNAC for FA (versus OBHA) was 100.0% (95% CI for sensitivity: 88.7–100.0%) and 75.0% (95% CI for specificity: 30.1–95.4%). The positive likelihood ratio for FNAC had a 95% CI which overlapped with 1.00. The negative likelihood ratio for FNAC was 0.00, and we were unable to compute the corresponding 95% CIs (No participants were FNAC negative and had FA on OBHA). The likelihood ratio for an indeterminate FNAC result was 0.86 (95% CI for likelihood ratio: 0.12–1.00). There were low diagnostic yields obtained for FNAC. The overall diagnostic accuracy of FNAC was 36.3% (95% CIfor overall diagnostic accuracy: 27.1–46.5%). The estimated prevalence of FA in the study population based on FNAC was 84.6% (95% CI for prevalence estimate: 75.8–90.6%). There were no uninterpretable test results for US, hence cross-tabulation for the US test is presented as a conventional 2x2 diagnostic accuracy table. The sensitivity and specificity of US for FA (versus OBHA) was 85.7% (95% CI for sensitivity: 77.9–93.5%) and 71.4% (95% CI for specificity: 47.8–95.1%). The positive and negative likelihood ratios for US had 95% CIs which did not include 1.00. The overall diagnostic accuracy of US for FA was 83.5% (95% CI for overall diagnostic accuracy: 74.6–89.8%). The estimated prevalence of FA in the study population based on US was 76.9% (95% CI for prevalence estimate: 67.3–84.4%).

## Discussion

Our findings from the CBE suggest that most of our (predominantly young) study population had benign breast disease with clinical characteristics commonly associated with FA^18^. Indeed, the OBHA confirmed all study participants to have benign breast disease, with the vast majority being diagnosed with FA.

While FNAC was performed for all participants in our study, interpretable results were only obtained for around one-third of FNAC tests performed. The high proportion of uninterpretable results for FNAC is not unique to our study^10^. The most common reason for uninterpretable FNAC results is inadequate specimen quality, and it is estimated that up to 28% of FNAC specimens might be inadequate^10^. By default this leads to the unnecessary requirement for OBHA, which has its own economic and patient-related consequences.

The poor yield for FNAC had negative consequences on measures of diagnostic accuracy for the test in our study. While the sensitivity and specificity for FNAC appear impressive in our study, this should be interpreted cautiously as the overall diagnostic accuracy of the test was poor. Other studies have also reported high sensitivity and specificity for FNAC, however these studies differ from ours in that they report high diagnostic accuracy for FNAC^9^,^10^. This can most likely be attributed to the level of experience the FNAC operators in our study possessed (as the primary operators were trainee physicians), which might have been much lower than that of the FNAC operators in other studies.^9^,^10^ It could also be that image-guided approaches, which were not available in our study, where used in these studies. The confidence intervals for the positive likelihood ratio and the likelihood ratio for an uninterpretable FNAC test included 1.00. If these likelihood ratios and confidence intervals are interpreted according to the recommendations of McGee^19^, we see that there is no change in the probability of FA based on these specific test results. The negative likelihood ratio for FNAC was 0.00, however as we were unable to compute confidence intervals for this likelihood ratio (possibly as a result of the size of our study population) it should also be interpreted with caution.

In our study, we found US to have a sensitivity and specificity similar to that obtained for unguided FNAC (based on overlapping confidence intervals). However, diagnostic accuracy was found to be significantly higher in US versus unguided FNAC (based on discrete confidence intervals for estimates for diagnostic accuracy). The lack of uninterpretable results from US tests may have contributed to the observed diagnostic accuracy. The confidence intervals for the positive and negative likelihood ratios for the US test did not include 1.00. If the recommendations of McGee^19^ are applied to these likelihood ratios, we find that there is a 20% increase in the probability of FA being present if the US test was positive and a 30% reduction in the probability of FA being present if the US test was negative. The findings of our study suggests that US could be very beneficial for the diagnosis of FA in this setting. This is also in agreement with the published literature which reports similar diagnostic accuracy for US^9^,^10^. However, there is still room for improvement in the overall diagnostic accuracy of 83.5% for US. Furthermore, a study by Kemp et al., found that one in ten women with benign findings on US had malignancy on histology^2^. In agreement with the recommendations of Kemp and colleagues^2^, we propose that “multiple readings” of US images should be performed, preferably independent readings by radiologists with varying levels of experience (ie. trainee and consultants) to rule out malignancy.

Although our study shows important individual findings for FNAC and US, there are also implications for the triple assessment in our setting. It appears that FNAC might be redundant when attempting to diagnose FA in a predominantly younger population. This finding is not unique. A British study by Yue et al., reported that FNAC can be omitted in patients <25 years old, particularly when CBE and US findings are normal or benign^20^. Specifically, the study of Yue and colleagues reported that a combination of CBE and US would have avoided needle biopsies in all but 3% of their study population^20^. While our study findings appear to support those of Yue et al., one must be reminded that our study is a pilot (hypothesis-generating) study, and therefore additional appropriately designed research is required before FNAC can be omitted from the FA diagnostic workup in younger SA women. This could result in reduced healthcare expenditure, as well as improved patient satisfaction with the FA diagnostic workup.

## Limitations

This patient sample was a consecutive/convenience sample of women with PBM who attended a surgical outpatient clinic at a regional academic SA hospital. As a non-probability sampling method was used, there is a risk of sampling bias in our study. Future research should include sampling methods which reduce the risk of selection bias. In addition, we report data from a single hospital/institution, and our findings might not be generalizable to other hospitals/settings. Future research should involve several hospitals/institutions. We do not present data on familial history of breast disease or HIV infection. However, these variables have not been shown to be associated with benign breast disease elsewhere^2^, and this validates their exclusion from this analysis. Our study sample size might appear modest (although adequate for the purposes of a pilot diagnostic study), however it is still larger than the sample size of the study by Kemp and colleagues. Our study population lacked participants with malignant disease and so the potential impact of malignancy on US and FNAC performance for FA diagnosis in our setting could not be investigated. We recommend further research with a larger sample size to adequately investigate the impact of malignancy on the diagnostic accuracy of US and FNAC for FA. Both FNAC and US are operator dependent. Due to resource constraints at our institution during the study period, all FNAC procedures were performed through the unguided approach. It is possible that the yield of FNAC tests with interpretable results might be increased through an image-guided approach in SA settings, though this would require further investigation. Finally, in our study only the US reports, and not the ultrasound images themselves, were available for interpretation. Therefore we were unable to present US imaging data outside that which was contained within the diagnostic reports. This limitation can only be addressed through prospectively designed studies, where additional copies of these imaging results are obtained for research purposes. While our study does have several limitations, we believe that it is an important study from a SA setting which provides preliminary information related to the diagnostic accuracy of FNAC and US for FA, particularly as we included uninterpretable results in our analysis. In general, these uninterpretable test results are often excluded or incorrectly assigned to other diagnostic outcome categories.

Breast ultrasonography had a much higher diagnostic accuracy for FA than unguided FNAC. Multiple readings might further improve the diagnostic accuracy of US. Our findings suggest that FNAC might not be necessary as part of the diagnostic workup for FA in some settings. We recommend further research be conducted to confirm our findings and address the limitations of our study.
